# Generation of clinical grade dendritic cells with capacity to produce biologically active IL-12p70

**DOI:** 10.1186/1479-5876-5-18

**Published:** 2007-04-12

**Authors:** Anke Zobywalski, Miran Javorovic, Bernhard Frankenberger, Heike Pohla, Elisabeth Kremmer, Iris Bigalke, Dolores J Schendel

**Affiliations:** 1 Institute of Molecular Immunology, GSF – National Research Center for Environment and Health, Munich, Germany; 2 Laboratory of Tumor Immunology, Ludwig Maximilian University, Munich, Germany; 3 Clinical Cooperation Group "Immune Monitoring", GSF – National Research Center for Environment and Health, Munich, Germany

## Abstract

**Background:**

For optimal T cell activation it is desirable that dendritic cells (DCs) display peptides within MHC molecules as signal 1, costimulatory molecules as signal 2 and, in addition, produce IL-12p70 as signal 3. IL-12p70 polarizes T cell responses towards CD4^+ ^T helper 1 cells, which then support the development of CD8^+ ^cytotoxic T lymphocytes. We therefore developed new maturation cocktails allowing DCs to produce biologically active IL-12p70 for large-scale cancer vaccine development.

**Methods:**

After elutriation of leukapheresis products in a closed bag system, enriched monocytes were cultured with GM-CSF and IL-4 for six days to generate immature DCs that were then matured with cocktails, containing cytokines, interferon-gamma, prostaglandin E2, and a ligand for Toll-like receptor 8, with or without poly (I:C).

**Results:**

Mature DCs expressed appropriate maturation markers and the lymph node homing chemokine receptor, CCR7. They retained full maturity after culture for two days without maturation cocktails and following cryopreservation. TLR ligand stimulation induced DCs capable of secreting IL-12p70 in primary cultures and after one day of coculture with CD40L-expressing fibroblasts, mimicking an encounter with T cells. DCs matured with our new cocktails containing TLR8 ligand, with or without poly (I:C), induced alloresponses and stimulated virus-specific T cells after peptide-pulsing. DCs matured in cocktails containing TLR8 ligand without poly (I:C) could also be loaded with RNA as a source of antigen, whereas DCs matured in cocktails containing poly (I:C) were unable to express proteins following RNA transfer by electroporation.

**Conclusion:**

Our new maturation cocktails allowed easy DC harvesting, stable maturation and substantial recoveries of mature DCs after cryopreservation. Our procedure for generating DCs is easily adaptable for GMP-compliance and yields IL-12p70-secreting DCs suitable for development of cancer vaccines using peptides or RNA as sources of immunizing antigens.

## Background

Dendritic cells (DCs) are superior antigen presenting cells that regulate the quality and the magnitude of immune responses [1]. DCs develop from bone marrow-derived progenitors and display an enormous plasticity with respect to lineage ontogeny, body location and functional diversity, with resultant differences in stimulatory capacity [2]. Recent discoveries describe mutual interference [3] and even distinct divisions of labor by different DC subtypes [4].

*In vivo*, immature- or intermediate-stage DCs patrol peripheral tissues to capture and process antigens [5]. Under the influence of local cytokines and danger signals, DCs undergo complex maturation processes and migrate to regional lymph nodes, where they form immunological synapses with T cells and present peptides derived from collected antigens in context with MHC class I or II molecules. They also provide costimulation and instructive signals (such as cytokines) that mirror the micromilieu under which they matured [6]. DCs can polarize immune responses, depending on the cytokines they secrete, yielding effector cells with different functional capacities. Thereby, DCs utilize unique features to induce responses in naïve T cells and ensure adequate effector functions [7]. Accordingly, DCs can serve as "natural adjuvants" in therapeutic vaccines for diseases with immunological dysfunction, including malignant disorders [8], infections and even allergic and autoimmune reactions [9].

Since DCs occur in only small numbers in blood, it is arduous to isolate them via antibody tagging for *ex vivo *manipulation [10]. *In vivo *targeting of DCs, as described recently via DC-specific molecules like DEC-205 [11] or DC-SIGN [12], would be highly advantageous but at present is complicated. Therefore, most current clinical applications depend on differentiation of bone marrow or peripheral blood DC progenitors using recombinant cytokines [13]. Numerous protocols exist to generate human DCs *in vitro *from CD34-positive cells or monocytic progenitors. Immature DCs (iDCs) are relatively easy to obtain in sufficient numbers from monocytes following culture with GM-CSF and IL-4. DC maturation can then be induced using inflammatory signals, such as tumor necrosis factor (TNF), interleukin-1 beta (IL-1β), or bacterial derivatives (e.g. lipopolysaccharide, lipoteichonic acid, or Ribomunyl), double-stranded RNA, interferons and/or prostaglandins [14].

There is substantial discussion whether DCs can function as tumor vaccines, based on the outcome of numerous clinical studies of different malignancies showing only limited rates of objective tumor regression in patients with advanced disease [15,16]. To date, most DC vaccination studies have utilized either iDCs or mature DCs (mDCs) not having a capacity to secrete biologically active IL-12. Thus, the question remains whether usage of DCs secreting this important cytokine would help to generate more effective cancer vaccines.

DCs producing IL-12p70 are desired for cancer vaccine development because of their leading role in promoting T helper 1 (Th1) cell polarization [17]. CD4^+ ^Th1 cells, in turn, support development of CD8^+ ^cytotoxic T lymphocytes, fostering the appropriate adaptive immune responses needed to combat minimal residual disease and control the outgrowth of malignant cells in tumor patients. IL-12p70-producing DCs can also support innate immunity through induction of natural killer (NK) cell proliferation. It has been shown that ligation of particular Toll-like receptors (TLRs) is a prerequisite to induce full maturation, enabling DCs to produce IL-12p70 [18].

Currently, a cocktail described by Jonuleit and coworkers is used commonly in clinical studies for DC maturation [19]. However, DCs matured with Jonuleit cocktail fail to produce IL-12p70. In contrast, Kalinski and coworkers described an alternative cocktail that provides mature DCs producing IL-12p70 [20]. In preliminary experiments, we experienced harvesting and handling problems of mDCs using this alternative cocktail. Therefore, we sought to establish new cocktails that would allow large-scale processing of clinical-grade mDCs with the capacity to secrete IL-12p70. To this end, we selected components of the Jonuleit cocktail that had a positive impact on DC maturation (TNF and IL-1β) while eliminating IL-6 which can inhibit IL-12p70 secretion. PGE2 was added at a low concentration to improve DC handling by reducing cell adherence. Finally, interferon-gamma (IFNγ) and the TLR8 ligand, R848 (Resiquimod), were included to induce IL-12p70 synthesis. Further amplification was achieved using poly (I:C) in one cocktail formulation. Since our vaccine strategies primarily envision using RNA as a source of tumor-associated antigens [21], it was also essential to establish that good protein expression would be obtained following RNA transfer into DCs generated in large-scale and matured with the new cocktails.

## Methods

### Leukapheresis and elutriation

To obtain monocytes as progenitor cells for generation of DCs, we used a closed bag system of elutriation using the ELUTRA instrument (Gambro BCT, Lakewood, CO, USA). After informed consent, healthy, untreated donors underwent 180 min leukapheresis (COBE Spectra; Gambro BCT) using a modified PBSC program: the separation factor was set to 700 with a collection rate of 0.8 mlmin and a target hematocrit of 1–2%. Harvested cells were analysed by an automatic blood counter (ACT Dif, Beckman Coulter, Krefeld, Germany) to set up parameters for the ELUTRA system.

Leukapheresis products were processed by ELUTRA, according to the manufacturer's instructions, by a method of counter-flow centrifugal elutriation using a fixed rotor speed (2400 rpm) and computer-controlled stepwise adjustment of medium flow rate, with rotor-off harvesting. Five liters of HANKs buffered salt solution (Biochrom, Berlin, Germany) with 1% human serum albumin (Octalbine^®^, Octapharma, Langen, Germany) were utilized for cell separation. ELUTRA processing yielded five fractions; enriched monocytes were present in fraction 5 and enriched lymphocytes were present in fraction 3. All fractions were characterized by automatic blood counter (ACT Dif) and flow cytometry.

### Generation of immature DCs from elutriated monocytes

Cells from fraction 5 were used directly for DC generation when CD14-positive monocytes represented more than 60% of cells detected by FACS. Fraction 5 cells were harvested from the collecting bag, washed once with PBS + 0.5% human serum and seeded at 35 × 10^6^/175 cm^2 ^cell culture flask (Nunc, Wiesbaden, Germany) in 35 ml DC medium containing RPMI 1640 with very low endotoxin (Biochrom), 1.5% human serum (pool of AB-positive adult males) (DRK-Blood Bank, Suhl, Germany) and 10 μg/ml gentamycin (Biochrom). They were cultivated for 6 days at 37°C, with 5% CO_2 _in a humidified atmosphere and on days 1, 3 and 6, cultures were supplemented with 100 ng/ml GM-CSF (Leukine^® ^by Berlex, Richmond, USA) and 20 ng/ml recombinant human IL-4 (R&D Systems, Wiesbaden, Germany) in 7 ml fresh DC medium.

### Maturation of DCs

Maturation was induced by adding different cocktails to iDCs on day 6, along with 7 ml fresh DC medium. Five cocktails were compared, including the Jonuleit [19] and Kalinski [20] cocktails that were described previously. The following components were utilized: TNF, IL-1β, IL-6 (R&D Systems); prostaglandin E2 (PGE2) (Minprostin^®^, Pharmacia/Pfizer, Erlangen, Germany); IFNα (Roferon A^®^, Roche, Welwyn Garden City, England); IFNγ (Imukin^®^, Boehringer Ingelheim, Ingelheim, Germany); double-stranded RNA (poly (I:C) and R848 (InVivogen, Toulouse, France). The exact composition of individual cocktails is provided in "Results". One flask of cells received only 7 ml fresh medium which served as the iDC control (data not shown). After incubation in maturation cocktails for 24 h, DCs were harvested by washing culture flasks twice in PBS + 0.5% human serum, with light shaking.

### Wash-out test

To analyze the stability of maturation, DCs were washed as described above and reseeded at 2.5–3 × 10^6^/9 ml fresh DC medium without any cytokines in 25 cm^2 ^culture flasks (Nunc). After approximately 44 h, DCs were harvested and phenotyped using selected antibodies.

### Cryopreservation of DCs

After harvesting and washing, 20–25 × 10^6 ^DCs were collected in 0.5 ml cold 20% human serum albumin (Octalbine^®^, Octapharma, Langen, Germany), gently mixed with freezing solution containing 10% glucose (Braun, Melsungen, Germany), 20% DMSO (CryoSURE^®^, WAK-Chemie, Dessau-Thornau, Germany) in 20% human serum albumin [22]. Cryotubes (Nunc), were stored overnight at -80°C in a special freezing container (Nalgene Nunc Intl. Corp. Rochester, N.Y., USA) and transferred into the gas phase of a liquid nitrogen container.

### FACS analysis of DCs

DCs were labeled with the following fluorescence-conjugated mouse monoclonal antibodies with appropriate isotype controls (clone X-40), CD14 (FITC, MΦP9), CD19 (FITC, clone: 4G7), CD86 (FITC, clone: 2331 FUN-1), CD80 (PE, clone: L307.4) (BD Biosciences); CD209 (PE, clone: DCN46) (Pharmingen, San Diego, USA); CD3 (FITC, clone: UCHT1, CD56 (FITC, clone: C5.9a), CD1a (FITC, clone: NA1/34) (Dako); HLA-DR (PE, clone: B8.12.2), CD40 (PE, clone: mAb89) and CD83 (PE, clone: HB15a) (Immunotech). CCR7 was detected using rat BLR-2 hybridoma supernatant medium (clone 8E8; E. Kremmer, GSF) with an IgG2a isotype control supernatant medium (EBNA-A2; clone R3, E. Kremmer) by incubation for 60 min, followed by washing and detection with a secondary mouse antibody against rat IgG conjugated with cyanin 5 (Jackson Immuno, West Grove, USA). Mature DCs electroporated with EGFP (enhanced green fluorescent protein)-encoding RNA were recultured with cocktails for 24 or 48 h, harvested, washed and green fluorescence was analyzed by flow cytometry within 1 h. To test vitality, DCs were pelleted and resuspended for 20 min in 7-amino-actinomycin D (Sigma-Aldrich, Deisenhofen, Germany) at a final concentration of 10 μg/ml in PBS + 2% fetal calf serum. After washing, 7AAD incorporation by dead cells were measured in the third channel of the FACS-Calibur flow cytometer.

### Signal-3 assay

DCs were cocultured with stimulating cells that mimicked T cells, as described previously [20,23]. Briefly, mDCs were reseeded in 96-well plates at 2 × 10^4^cells/well and incubated with 5 × 10^4 ^cells/well of mouse fibroblast L-cells stably transfected to express human CD40-Ligand (CD40L) [24]. As controls, DCs and L-cells were cultured alone in medium without cytokines. After 24 h, plates were centrifuged and supernatants of eight replicate wells were pooled for analysis of IL-12p70 and IL-10 by ELISA.

### ELISA (IL-12p70/IL-10)

IL-12p70 and IL-10 secreted by DCs during the maturation process (primary DCs) and in the signal-3 assay were detected by standard ELISA, utilizing antibody duo-sets (R&D Systems), according to the manufacturer's instructions. Colorimetric substrate reactions using tetramethyl-benzidine and H_2_O_2 _were stopped with H_3_PO_4_, measured at 450 nm with wavelength correction at 620 nm, and analyzed by "easy fit" software (SLT, Crailsheim, Germany).

### Mixed lymphocyte reaction

Mature DCs were washed twice in PBS + 0.5% human serum, irradiated with 40 Gy and 1 × 10^4^cells/well plated in 96-well round bottom microplates (Nunc) in RPMI 1640 + 1.5% human serum. Cryopreserved cells from ELUTRA fraction 3 (lymphocyte-enriched fraction) were used as responder cells at a concentration of 1 × 10^5 ^cells/well. As controls for T cell activation, third party mononuclear cells (MNCs) from buffy coats of five unrelated donors were pooled and used as stimulating cells after irradiation with 40 Gy. T cell proliferation was also controlled by addition of IL-2 (Proleukin^®^, Chiron, Emeryville, CA, USA) at 50 IU/ml, or 5 IU/ml in combination with phytohaemagglutinin (PHA) at 10 μg/ml (Sigma-Aldrich, Deisenhofen, Germany). After 6 days, cells were pulsed with 0.5 μCi/well ^3^H-thymidine (Amersham-Pharmacia, Freiburg, Germany) and radioactivity measured after 24 h.

### ELISPOT assay of virus-specific T cell activation

For activation, lymphocytes from ELUTRA fraction 3 were plated at 1 × 10^6 ^cells/well with 1 × 10^5 ^viral peptide-loaded DCs in 24-well plates, in RPMI 1640 medium with 10% human serum; 30 IU/ml IL-2 was added at d3 and lymphocytes harvested at d7. HLA-A*0201-binding peptides included: CMVpp65_495–503 _(NLVPMVATV), EBV-BMLF1_280–288 _(GLCTLVAML), influenza M1 protein_58–66 _(GILGFVFTL) or the CEF pool (PANATecs GmbH, Tuebingen, Germany) containing two additional peptides, EBV-LMP-2_426–434_(CLGGLLTMV) and influenza RNA polymerase PA_46–54 _(FMYSDFHFI). *In vitro *activated T cells and autologous monocytes plus CEF peptides were incubated in RPMI 1640 medium containing 2 mM L-glutamine, 1 mM sodium pyruvate, penicillin/streptomycin (100 U/ml), 10% human AB serum (BioWhittaker, Verviers, Belgium) and 20 IU/ml IL-2 at 37°C with 5% CO_2 _for 24 h. IFNγ-ELISPOT analysis was performed as described [25,26], with the exception that antibody precoated PVDF plates (Mabtech AB, Nacka, Sweden) and streptavidin-alkaline phosphatase and a ready-to-use BCIP/NBT-plus substrate solution (Mabtech) were used for detection. Spots were counted using the AID reader system ELR03 with 3.2.3 software (AID Autoimmun Diagnostika GmbH, Strassberg, Germany).

### EGFP-RNA transfection into DCs

EGFP-RNA was produced *in vitro *and electroporated into mDCs at 24 h, as described previously [27,28] with the exception that each 0.4 cm electroporation cuvette contained a total volume of 300 μl, including 8 μg of EGFP-RNA and 3× 10^6 ^DCs. After electroporation, mDCs were returned to their original maturation media and incubated in a 24-well plate at 37°C and 5% CO_2 _for 24 or 48 h before flow cytometric analysis.

## Results

### Establishment of primary DC cultures using elutriated monocytes

**Figure 1:**
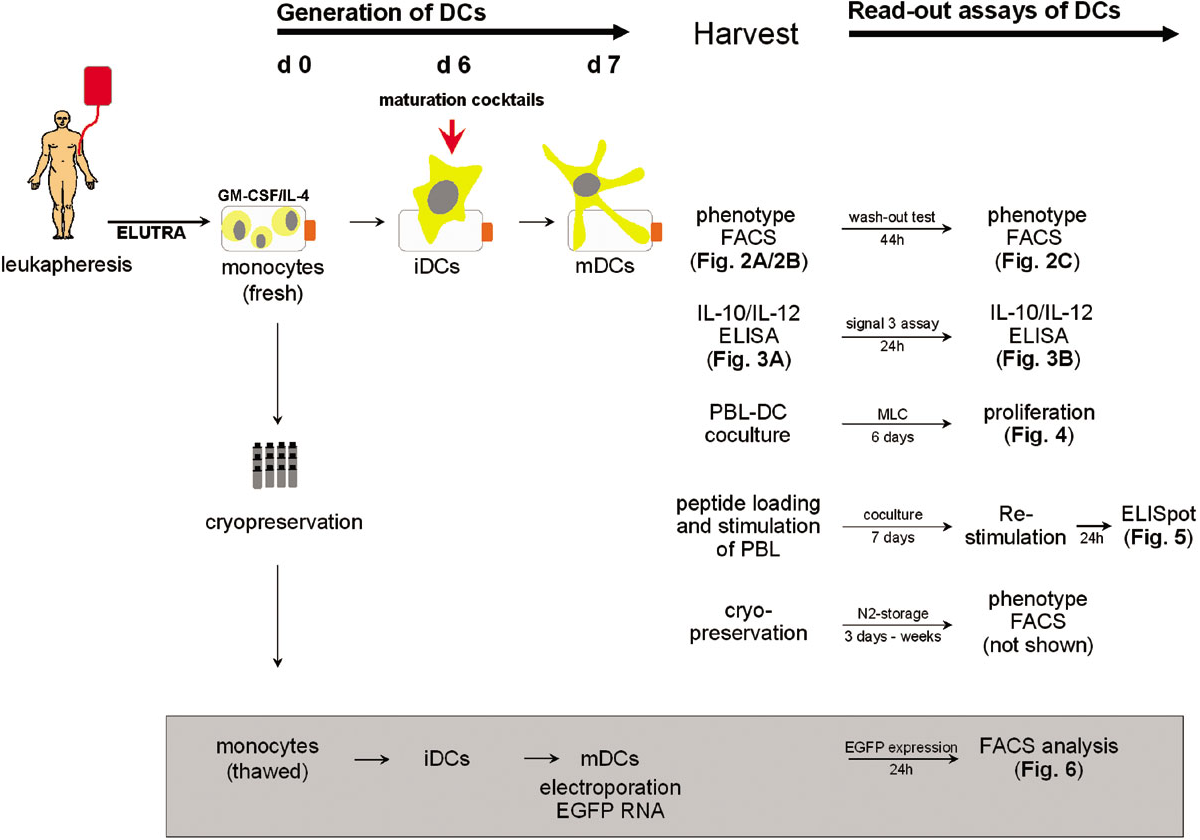
**Time-line of experimental setup using monocytes derived from one representative donor**. Monocytes were prepared from a leukapheresis by elutriation on day 0 and cultured for 6 days with GM-CSF and IL-4 to produce iDCs, which were then incubated with different maturation cocktails. After 24 h, mDCs were harvested and washed twice, phenotypes determined by FACS and aliquots were cryopreserved. The primary culture supernatants were collected to assess IL-12p70 and IL-10 by ELISA. Samples of the different DC populations were cocultured with fibroblast L-cells (signal-3 assay) for an additional 24 h and supernatants collected once again for IL-12p70 and IL-10 measurements. Mixed lymphocyte cultures were established using autologous and allogeneic lymphocytes (fraction 3 of elutriated leukaphesis cells, cryopreserved on day 0) as responding cells and DC1–DC5 cells as stimulating cells. Tritiated-thymidine incorporation into dividing cells was measured during the final 24 h of a 7-day coculture. Mature DC1–DC5 cells that were harvested and washed on day 7 were loaded with CEF peptides and used as stimulating cells for autologous lymphocytes (fraction 3 cells after elutriation, cryopreserved on day 0). Lymphocytes and the various DC populations were cocultured for 7 days, washed and restimulated with autologous monocytes (fraction 5 cells, cryopreserved on day 0), with or without CEF peptides. IFNγ secretion was assessed in a standard ELISPOT analysis 24 h later. DCs cryopreserved on day 7 were thawed and reassessed for phenotype after storage in liquid nitrogen. Cryopreserved monocytes (fraction 5 from day 0) were thawed and used to generate new mDCs which were loaded with EGFP RNA by electroporation on day 7. Flow cytometry to detect percentages of positive cells and intensity of fluorescence was performed at 24 h.

**Figure 2:**
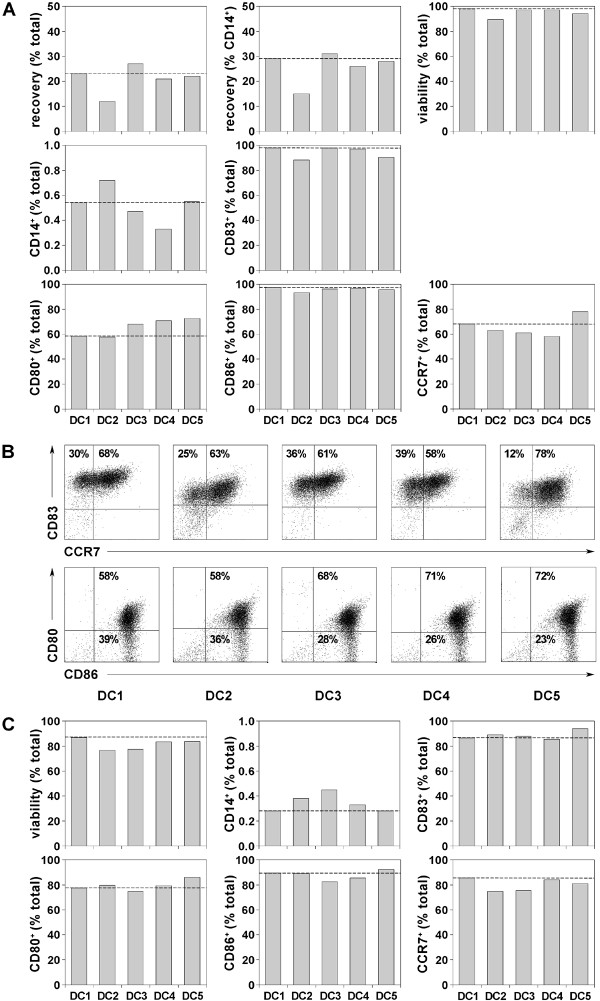
**Generation of stable, mature DCs using different maturation cocktails**. (A) Percentages of DCs harvested after primary cell culture (6 d differentiation + 24 h maturation) calculated on total seeded cells (mononuclear cells) or CD14-positive monocytes detected by FACS and manual counting. DC populations DC1–DC5 were matured in different cocktails as listed in Table 1. Viability was detected by 7AAD exclusion measured by flow cytometry in FL-3 of the FACS-Calibur and viable cells are expressed as percentages of total cells. Percentages of cells expressing various surface markers were determined by flow cytometry using the antibodies specified in "Methods", including CD14 (a monocyte marker), CD83 (a marker of mDCs), CD80 and CD86 (costimulatory molecules) and chemokine receptor 7 (CCR7 = CD197) as an indicator for DC migratory potential into lymph nodes. Data are expressed as percentages of total cells with acquisition of 1 × 10^4 ^events. CCR7 values presented here (Fig. 2A) represent the percentages in histograms overlayed by IgG2a isotype control, although generated from the same FACS stain they are slightly different to the CCR7 values shown as dot plots in Figure 2B. Broken lines indicate marker levels for DCs matured with DC1 (Jonuleit) cocktail. (B) Representative dot plots of DC1–DC5 populations showing percentages of cells positive for CD83 versus CCR7 and CD80 versus CD86. (C) DCs were washed free of maturation cytokines and cultured an additional 44 h in culture medium without cytokines. Viability was determined by 7AAD incorporation. Percentages of CD14, CD83, CD80, CD86 and CCR7 were determined as described above. Broken lines indicate marker levels for DCs matured with DC1 (Jonuleit) cocktail.

Monocytic progenitor cells were obtained by elutriation of leukapheresis products of healthy donors. In order to assess a variety of parameters, DCs obtained from monocytes of one donor were used throughout an extensive series of experiments examining phenotype and function; one full set of these experiments is shown here. (Fig. 1). The stepwise procedures and characterization of primary DCs generated from this donor are representative of results obtained with different donors in independent experiments. ELUTRA fraction 5 from this donor contained 80.6% CD14-positive cells and the following contaminants: 2.9% CD3-, 2.2% CD56-, 1.5% CD19- and 7.7% CD67-positive cells (data not shown) and therefore was of appropriate quality to prepare DCs. Following generation of iDCs using GM-CSF and IL-4, cells were matured by incubation with the various maturation cocktails listed in Table 1. Recoveries of mDCs, based on total numbers of seeded cells and starting numbers of CD14-positive monocytes, were lower in DC2 (Kalinski) cocktail compared to DC1 (Jonuleit) cocktail (Fig. 2A), due to cell loss through strong plastic adherence of DC2 cells. DC viability ranged from 88.8% to 97.9%, with the lowest value for DC2 cells. The monocyte marker CD14 disappeared and the mDC marker CD83 appeared, thereby demonstrating that all populations contained highly mature DCs (Fig. 2A, 2B). High expression of costimulatory molecules, like CD80 and CD86, reflected the typical phenotype of mDCs, and chemokine receptor CCR7 (CD197) expression indicated that the majority of DCs had migratory potential for lymph nodes (Fig. 2A, 2B). Table 2 summarizes the phenotypic analyses of primary DCs prepared in independent experiments from different donors matured in the various cocktails, demonstrating that there were no substantial differences with respect to surface marker expression among the mDCs.

**Table 1:** Cocktail compositions used for DC maturation

**DC population**	**Cocktail**^a^	**Inflammatory cytokines/interferons**	**Other additives**	**TRL-ligands**	**Comments**
DC1	Jonuleit	TNF, IL-1β, IL-6	PGE2	-	no TLR ligands
DC2	Kalinski	TNF, IL-1β, IFNα, IFNγ	-	poly (I:C)	TLR3/non-TLR ligand
DC3	Cocktail 3^b^	TNF, IL-1β, IFNγ	PGE2	R848	TLR8 ligand, less IFNγ and PGE2
DC4	Cocktail 4^b^	TNF, IL-1β, IFNγ	PGE2	R848	TLR8 ligand
DC5	Cocktail 5^b^	TNF, IL-1β, IFNγ	PGE2	R848, poly (I:C)	TLR3/non-TLR, TLR8 ligands

^a ^The following concentrations were used for the individual cocktails: Jonuleit: 10 ng/ml TNF, 10 ng/ml IL-1β, 15 ng/ml IL-6 and 1 μg/ml prostaglandin E2; Kalinski: 10 ng/ml TNF, 10 ng/ml IL-1β, 3000 IU/ml IFNα, 1000 IU/ml IFNγ and 20 ng/ml poly (I:C); Cocktail 3: 10 ng/ml TNF, 10 ng/ml IL-1β, 1000 IU/ml IFNγ, 1 μg/ml R848 and 100 ng/ml prostaglandin E2; Cocktail 4: 10 ng/ml TNF, 10 ng/ml IL-1β, 5000 IU/ml IFNγ, 1 μg/ml R848 and 250 ng/ml prostaglandin E2; Cocktail 5: 10 ng/ml TNF, 10 ng/ml IL-1β, 5000 IU/ml IFNγ, 1 μg/ml R848, 250 ng/ml prostaglandin E2 and 20 ng/ml poly (I:C).

^b ^These represent compositions first reported here.

**Table 2:** Characteristics of primary DCs following maturation with different cocktails

**Parameter**		**DC1**	**DC2**	**DC3**	**DC4**	**DC5**
Recovery (% seeded cells)	N*	4	4	3	2	2
	m^+^	32.8	17.6	19.0	17.9	18.1
	std^#^	18.7	9.2	5.5	3.0	6.0
						
Recovery (% CD14^+^cells)	N*	4	4	3	3	2
	m^+^	42.6	22.2	25.4	24.0	25.5
	std^#^	22.0	9.7	6.2	2.8	3.1
						
% viable cells (7AAD-neg cells)	N*	5	5	4	5	3
	m^+^	97.5	88.9	96.0	96.0	94.0
	std^#^	0.9	3.7	1.6	1.2	1.4
						
% CD14^+^cells	N*	5	5	4	5	3
	m^+^	2.2	2.5	1.6	2.5	3.1
	std^#^	2.2	3.0	0.9	3.1	4.1
						
% CD83^+ ^cells	N*	5	5	4	5	3
	m^+^	94.2	82.3	95.1	90.6	92.4
	std^#^	3.0	7.1	2.3	10.7	1.7
						
% CCR7^+ ^cells	N*	5	5	4	5	3
	m^+^	79.7	67.5	75.2	74.4	85.2
	std^#^	7.2	5.6	8.7	11.8	0.7
						
% CD80^+ ^cells	N*	5	5	4	5	3
	m^+^	80.7	70.8	83.2	85.5	85.3
	std^#^	12.5	10.9	10.7	8.9	11.0
						
% CD86^+ ^cells	N*	5	5	4	5	3
	m^+^	95.1	89.6	94.2	94.7	95.8
	std^#^	2.2	4.5	2.2	2.1	0.8

* N = number of independent donors

^+ ^m = mean

^#^std = standard deviation

To determine if maturation was stable, the same mDCs were evaluated after 44 h of re-culture in medium without cytokines (Fig. 2C). As expected, some viability was lost, nevertheless more than 80% viable cells were recovered in all cases. No reversal of differentiation was observed since all DCs remained CD14-negative and retained high levels of CD83, CD80, CD86 and CCR7. Thus, all cocktails induced stable maturation of DCs. The stability of maturation, as assessed for DCs prepared from different donors in independent experiments is summarized in Table 3, revealing that DC3, DC4 and DC5 cells were comparable to cells matured in DC1 (Jonuleit) and DC2 (Kalinski) cocktails.

**Table 3:** Characteristics of DCs from different donors following wash-out of maturation cocktails and cryopreservation

**Parameter**		**DC1**	**DC2**	**DC3**	**DC4**	**DC5**
***Wash-out***						
% viable cells	N*	5	5	4	5	2
	m^+^	93.4	81.4	84.9	87.4	80.2
	std^#^	4.2	4.2	5.0	3.6	4.8
						
% CD14^+ ^cells	N*	5	5	4	4	2
	m^+^	0.9	4.7	0.8	2.3	3.6
	std^#^	1.0	7.3	0.4	3.3	4.7
						
% CD83^+ ^cells	N*	5	5	4	5	2
	m^+^	91.7	79.3	86.5	86.1	88.2
	std^#^	3.0	8.4	6.8	6.5	8.2
						
% CCR7^+^cells	N*	5	5	4	5	2
	m^+^	89.4	71.4	81.7	86.1	76.1
	std^#^	4.3	15.1	6.6	4.6	6.9
						
***Cryopreservation***						
						
% viable cells	N*	3	2	2	3	2
	m^+^	86.5	60.6	81.8	84.5	64.2
	std^#^	4.0	4.4	2.1	1.8	14.9
						
% CD14^+ ^cells	N*	3	2	2	3	2
	m^+^	1.6	0.4	0.5	1.9	1.8
	std^#^	2.2	0.3	0.2	2.5	2.1
						
% CD83^+^cells	N*	3	3	2	3	2
	m^+^	95.8	84.9	97.5	96.1	95.3
	std^#^	2.1	7.3	0.0	1.1	3.9
						
% CCR7^+ ^cells	N*	3	2	2	3	2
	m^+^	81.3	76.3	80.7	81.6	84.5
	std^#^	6.6	7.4	2.5	6.3	0.2
						
% CD80^+^cells	N*	3	2	2	3	2
	m^+^	80.6	71.6	87.8	90.4	82.7
	std^#^	5.6	3.8	4.4	0.9	3.7
						
% CD86^+ ^cells	N*	3	2	2	3	2
	m^+^	96.3	94.8	96.9	96.0	93.5
	std^#^	2.7	0.8	0.9	1.4	6.8

* N = number of independent donors

^+ ^m = mean

^#^std = standard deviation

### Recovery of DCs after cryopreservation

Cryopreserved mDCs were stored in the gas phase of liquid nitrogen and subsequently thawed for assessment of viability and phenotype. Viabilities of over 80% were found with mature DC1, DC3 and DC4 populations. In contrast, the viabilities of DC2 and DC5 cells, both matured in cocktails containing poly (I: C), dropped to 63% and 74%, respectively. High expression of CD83, CD80, CD86 and CCR7 was retained on all DCs and very few CD14-positive cells were detected (data not shown). Therefore, mDCs could be recovered after cryopreservation with a maturation status suitable for clinical use, as shown in comparisons of DCs prepared from additional donors in independent experiments (Table 3).

### IL-12p70 and IL-10 release by mDCs

**Figure 3:**
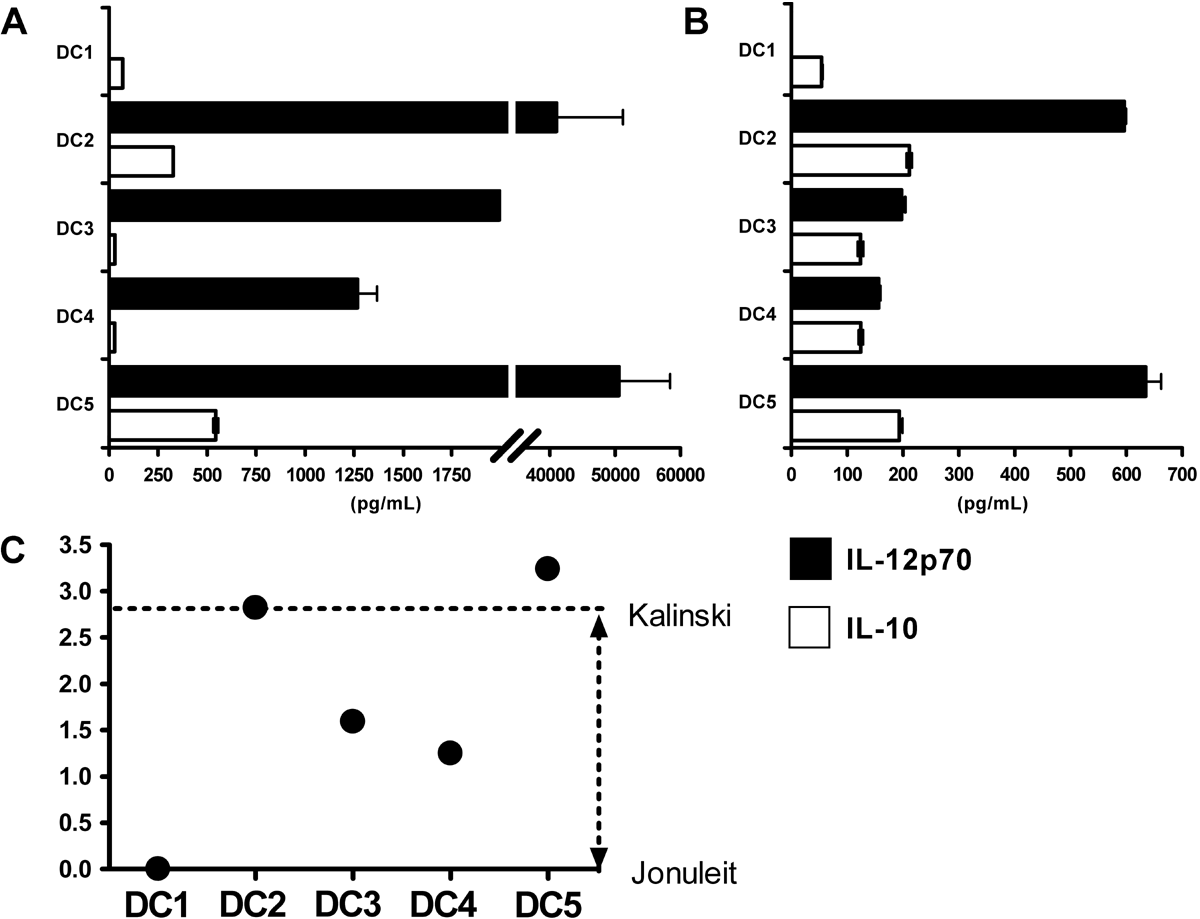
**Production of IL-12p70 and IL-10 by DCs matured using different cocktails**. Immature DCs were cultured with different maturation cocktails and the amounts of IL-12p70 and IL-10 were determined by standard ELISA. Filled bars indicate IL-12p70 and empty bars IL-10, respectively. Cytokine content was measured (A) in supernatant medium of primary maturation cultures after 7 d and (B) in supernatant medium of cultures containing washed mDCs and CD40L-transfected fibroblasts following coculture for 24 h, representing a signal-3 assay as described in "Methods". (C) The quotients of IL-12p70/IL-10 were determined for the DC populations matured in different cocktails, based on the pg/ml values of the signal-3 assay. For calculation of quotients, it was assumed that IL-12p70 and IL-10 have theoretically equal biological potential. Filled circles indicate quotients ranging from 0 (DC1 cells in Jonuleit cocktail) to 3.2 (DC5 cells in cocktail 5). The values of DCs matured in DC1 (Jonuleit) versus DC2 (Kalinski) cocktails are indicated by broken lines.

Our primary goal was to create cocktails that yielded mDCs with a capacity to produce IL-12p70 that could be easily produced in large-scale. Figure 3A shows secretion of biologically active IL-12p70 and IL-10 by primary mDCs into culture supernatant media. DC1 cells did not produce any IL-12p70, whereas DC2–DC5 cells secreted IL-12p70 in the ng/ml ranges. As a second assay, mDCs were coincubated with fibroblast L-cells transfected to express human CD40L, thereby mimicking an encounter of DCs with T cells (Fig. 3B). CD40L-stimulation induced renewed IL-12p70 secretion from DC2 and DC5 cells and to a lesser extent from DC3 and DC4 cells, whereas no IL-12p70 was found with DC1 cells. Because IL-10 counteracts IL-12, we calculated the quotient of IL-12p70/IL-10 for all mDCs (Fig. 3C). DC1 cells had the lowest quotient, whereas DC2 and DC5 cells showed the highest quotients in this experiment. DCs matured with cocktails DC3 and DC4 also secreted more IL-12p70 than IL-10 and were superior to DC1 cells in this respect. Variations were noted in the capacity of DCs derived from different donors to produce these cytokines (Table 4). Nevertheless, DC2–DC5 cells were always superior to DC1 cells with respect to IL-12p70/IL-10 values.

**Table 4:** Signal-3 assay of cytokine production by DCs matured in different cocktails

**Parameter**		**DC1**	**DC2**	**DC3**	**DC4**	**DC5**
	N*	7	5	4	7	3
						
IL-12p70(pg/ml)	m^+^	2.6	432.7	346.4	181.4	466.2
	std^#^	4.5	313.3	529.3	290.9	318.3
						
IL-10(pg/ml)	m^+^	25.3	153.0	61.5	52.9	157.3
	std^#^	34.6	124.5	51.7	45.1	64.3
						
IL-12p70/IL-10(quotient)	m^+^	0	5.1	4.2	5.2	3.9
	std^#^	0	7.2	6.6	7.4	3.8

* N = number of independent donors

^+ ^m = mean

^#^std = standard deviation

### Allostimulatory capacity of DCs

**Figure 4:**
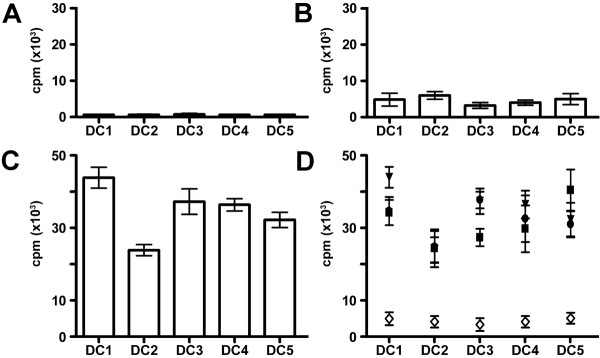
**Analysis of allostimulatory capacity of different DCs in mixed lymphocyte reactions**. (A) Negative controls of proliferation of irradiated (40 Gy) DCs alone. (B) Proliferation of autologous T cells stimulated by DC1–DC5 populations. (C) Proliferation of one representative allogeneic T cell responder stimulated by DC1–DC5 cells. Note that the y-axis is different for autologous and allogeneic T cell responses. (D) Summary of proliferation data of three independent allogeneic T cell responders in comparison to autologous T cells stimulated by DC1–DC5 cells.

To evaluate DC function, we utilized mixed lymphocyte reactions employing mDCs as stimulating cells with autologous and allogeneic T cells. T cell proliferation against third party cells and PHA plus IL-2 or IL-2 alone served as positive controls, demonstrating that both autologous and allogeneic T cells had strong proliferative potential (data not shown). Irradiated DCs alone did not proliferate (Fig. 4A), verifying that the assay only detected T cell responses. As expected, autologous T cells proliferated less well than allogeneic T cells following DC stimulation (Fig. 4B versus 4C). Figure 4D summarizes responses of three independent allogeneic responder T cells versus autologous T cells with the various DCs of the selected donor. As expected, all the different mDCs induced strong alloresponses due to MHC disparity however responses were somewhat lower using DC2 cells. Table 5 demonstrates that DCs matured in all five maturation cocktails had substantial allostimulatory capacity in three independent experiments.

**Table 5:** Stimulation of autologous and allogeneic PBLs using DCs matured in different cocktails

**Parameter**		**DC1**	**DC2**	**DC3**	**DC4**	**DC5**
DCs only control (cpm)	N*	3	3	2	3	2
	m^+^	636.3	639.5	659.7	518.5	607.7
	std^#^	98.7	145.3	207.7	165.5	63.4
						
Autologous PBLs(cpm)	N*	3	3	2	3	2
	m^+^	3704.9	3291.9	3498.4	5490.7	2864.1
	std^#^	2602.9	2619.3	4611.8	6022.2	3012.1
						
Allogeneic PBLs(cpm)	N*	7	7	5	7	5
	m^+^	37531.5	28635.2	38513.0	36918.9	37052.2
	std^#^	4313.8	5661.2	7935.8	11058.8	5142.4

* N = number of independent donors

^+ ^m = mean

^# ^std = standard deviation

### Induction of IFNγ secretion by T cells following coculture with peptide-pulsed DCs

**Figure 5:**
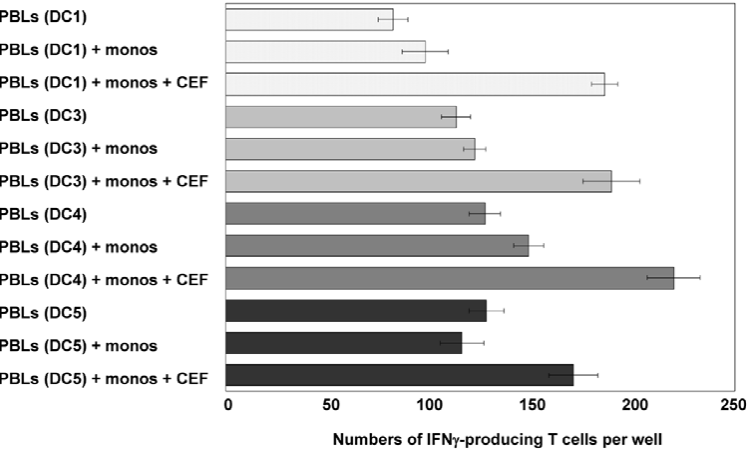
Response of autologous lymphocytes from an HLA-A*0201-positive donor responding to virus-peptide pulsed DCs. T cell responses were assessed in an IFNγ-ELISPOT experiment using peripheral blood lymphocytes (PBLs: T cell enriched ELUTRA fraction 3 = 54.8% CD3 positive cells) that were first activated for 7 d with mature peptide-pulsed DCs and then restimulated for 24 h with monocytes plus CEF peptides. For the ELISPOT analyses, 4 × 10^3 ^autologous *in vitro *activated lymphocytes were stimulated with 2 × 10^3 ^monocytes together with the CEF peptide pool. The mean ± S.D. was calculated for triplicate wells. Note: Due to insufficient recoveries, lymphocytes activated by DC2 cells were not included in the assay. Because of limitations in HLA-A2 subtyped donors available for leukapheresis, this experiment was only included once in the full DC evaluation protocol.

The capacity of the same DC populations to present peptides was tested using a set of virus-specific peptides derived from cytomegalovirus, Epstein-Barr virus, and influenza virus (CEF peptides) (Fig. 5). Autologous lymphocytes of ELUTRA fraction 3 of the HLA-A*0201-positive DC donor were stimulated for 7 days with peptide-pulsed DCs matured in the various cocktails. Activated lymphocytes recovered from the cocultures with DC1, DC3, DC4 and DC5 cells were then restimulated for 24 h with autologous monocytes, which were cryopreserved following elutriation on day 0 (Fig. 1), with or without addition of CEF peptides. It was not possible to assess the stimulating capacity of DC2 cells in this assay due to insufficient cell recoveries. The capacity of the DC-activated lymphocytes to secrete IFNγ in response to peptide-restimulation was assessed in a standard ELISPOT assay. Increased responses were seen in the presence of CEF peptides in all cases, demonstrating that DCs matured in our cocktails could present peptides and reactivate memory T cells with efficiencies comparable to DCs matured in DC1 (Jonuleit) cocktail. It should be noted here that this capacity has been demonstrated elsewhere for DC2 cells (matured in Kalinski cocktail) [20].

### Expression of protein following RNA transfer into DCs by electroporation

**Figure 6:**
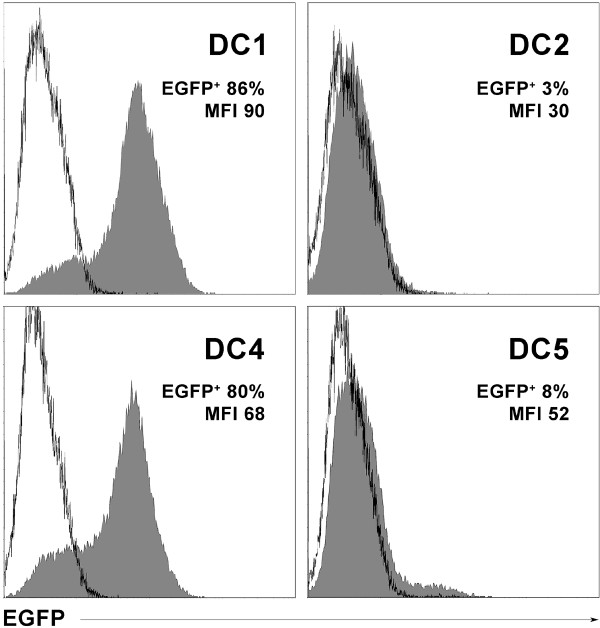
**Expression of EGFP in DCs transfected with EGFP-encoding *in vitro *transcribed RNA**. Flow cytometry histogram overlays show EGFP expression following RNA transfer into mDCs on day 7 (filled curves) 24 h after electroporation and corresponding untransfected DCs (empty curves) as negative controls. DCs were matured in the four cocktails indicated, RNA was introduced by electroporation, the DCs were returned to their corresponding media containing maturation cocktails and harvested for flow cytometry 24 h later (day 8). Numbers indicate the percentages of EGFP-positive DCs and their mean fluorescence intensities. These data are representative of two independent experiments with measurements at 24 and 48 h.

RNA is an attractive alternative source to provide antigens to DCs, whereby a whole protein is directly translated in the cytosol and made available to DCs for peptide processing and presentation, thereby bypassing the need to know specific MHC-binding peptides. To test the capacity of DCs to express protein after loading with *in vitro *transcribed RNA, we analyzed EGFP expression by flow cytometry after transfer of corresponding RNA. Mature DCs were generated from cyropreserved monocytes obtained by leukapheresis and elutriation according to the time-line shown in Figure 1. Due to limitations in cell numbers, DC3 cells were not included because cocktail DC3 was identical to cocktail DC4, except for lower amounts of IFNγ and PGE2 (Table 1). On day 6, the different maturation cocktails were added to the iDCs and on day 7, RNA was introduced into the mDCs via electroporation. In previous studies using DCs matured in DC1 (Jonuleit) cocktail, EGFP expression was found to peak 12–24 h following RNA transfection and expression was stable for 48 h [28]. Therefore, percentages of EGFP-positive cells and mean fluorescence intensities (MFI) were measured 24 and 48 h after electroporation. DC1 and DC4 cells expressed EGFP whereas DC2 cells did not express EGFP and DC5 cells expressed no (Fig.6) or only very low levels of EGFP (data not shown). This same pattern was seen at 48 h (data not shown). Poly (I:C), which was a component of cocktails DC2 and DC5 was not present in cocktails DC1 and DC4. DC2 (Kalinski) cocktail also contained IFNα, which was not included in any other cocktail. Interestingly, we found elsewhere that DCs matured with IFNα alone also failed to express EGFP protein following RNA transfer [21].

## Discussion

DC-based immunotherapy has been proven to be safe with minimal side-effects and has shown some clinical efficacy in patients with metastatic melanoma [29-31]. Induction of effective immune responses is dependent upon proper maturation of DCs [32]. Such DCs provide three interactive functional signals within an immunological synapse of appropriate duration [33-35]. Signal 1 provides the specificity for an immune response through DC presentation of MHC-peptide complexes to responding T cells. Secondly, mature DCs provide positive amplification of signal 1 transduction via costimulation (signal 2) through molecules such as CD80, CD86 and CD40. Thirdly, DCs translate the environmental patterns under which they matured into the quality of effector cell responses by secreting various cytokines (signal 3) that regulate, for example, T helper cell polarization. Thus, IL-12p70 secretion leads to Th1 responses which, in turn, support the induction of CTLs, macrophages or DTH reactions via production of IL-2, IFNγ and TNF. Alternatively, IL-10 or TGFβ support Th2 responses that impact on antibody production, mast cell degranulation and eosinophilia via secretion of IL-4, IL-5, IL-10, IL-13 [36,37]. Optimal adaptation of monocyte-derived DCs generated *in vitro *for cancer vaccines needs to take these aspects into account in order to improve treatments for malignant diseases.

We established a closed bag system of elutriation using leukapheresis products to obtain monocytic progenitor cells for DC generation. The method required some finesse, particularly regarding the adaptation of the leukapheresis collection mode to assure low erythrocyte contamination. During more than 20 elutriation runs (data not shown), we noted individual variations based on granulocyte levels; if these were high in peripheral blood, granulocytes were often retained in fraction 5 due to their similar size characteristics with monocytes. Removal of platelets in fraction 1 was highly advantageous and was not achieved with other monocyte-enrichment methods, such as Ficoll-gradient centrifugation or magnetic-bead separation (data not shown). The elutriation process with the ELUTRA system proved to be generally feasible for monocyte isolation, as also described by other groups [38], and is easily adapted to GMP guidelines.

We evaluated several maturation cocktails for clinical use with monocyte-derived iDCs generated using GM-CSF and IL-4. DCs generated from the same leukapheresis products using either elutriation or Ficoll separation and subsequent plastic adherence had identical phenotypes 24 h after maturation with DC1 (Jonuleit) cocktail (data not shown). This revealed that monocyte-derived DCs prepared by elutriation were of the same quality.

RPMI 1640 medium based on ultra-pure water suitable for patient injection and very low endotoxin content was used for DC cultures. Protein supplementation was made with 1.5% specialized human male AB-serum, pooled from at least 20 donors. Sera were combined only after each single donor was tested extensively for infection markers at the time of donation and 4 months later. Additionally, the serum pool was demonstrated to be negative for IL-12p70 and IL-10 (data not shown). According to standard guidelines for blood products, this serum pool should be suitable for the regulatory authorities. In contrast to other approaches utilizing autologous plasma/serum [31], we considered that tumor patient-derived proteins could be problematic in some instances due to the presence of individual inhibitory cytokines. Use of a standardized serum pool of healthy donors mostly free of inhibitory cytokines would eliminate another variable in small clinical trials. A complete synthetic medium would be desirable but several commercially available serum-free alternatives were not satisfactory in our hands.

Further, we created new maturation cocktails and compared them with DC1 (Jonuleit) cocktail [19] and the recently described DC2 (Kalinski) cocktail [20]. We also evaluated several synthetic TLR7/8 ligands such as Resiquimod (R848), Imiquimod (R837), Loxoribine, or poly U-rich single-stranded RNA. As reported by Napolitani and co-workers [39], we found the imidazoquinilone-derivate R848 to be superior (data not shown), despite positive influences on DCs by Imiquimod[40] and Loxoribine [41]reported also by others.

Based on these comparisons, we developed a new cocktail based on TNF, IL-1β, IFNγ, PGE2 and R848. Because of a dose-dependent IL-12-inhibitory potential, the concentration of PGE2 was lowered compared to DC1 (Jonuleit) cocktail but was retained in order to support induction of CCR7 expression and improve harvesting feasibility through reduced plastic adherence of the matured DCs. To further amplify IL-12-inducing potency, poly (I:C) was added to cocktail DC5. All cocktails induced high levels of CD83 expression on DCs cultured for 7 days. Costimulatory molecules were also high on all cells and CCR7 was present on over 60% of cells. DCs matured with the DC2 (Kalinski) cocktail showed somewhat lower viabilities due to their strong adherence and presence of elongated dendritic veils that hindered harvesting. In contrast to published results [20], this appeared as a disadvantage in our system, which was most apparent with respect to functional assays and DC cryopreservation. Stability of maturation is another important characteristic for clinically applied DCs because patients with malignant diseases often have high serum levels of inhibitory cytokines (e.g. IL-10, TGF-β or IL-6). These cytokines may reverse DCs to an immature status, which could then tolerize a patient's immune system towards the immunizing tumor antigens. DCs matured with all cocktails showed stable CD83 expression for two days after removal of cytokines, suggesting irreversible maturation had occurred during this time period in all DC populations.

Legal requirements for extensive cleaning and disinfection of a GMP clean room between the handling of cells of different patients led us to develop a mode of DC generation that would yield high numbers of cells that could be cryopreserved in a single batch in order to avoid repetitive cultures for individual patients. It also has been shown that it is possible to freeze mature DCs even after antigen loading [22]. DCs matured with all the different cocktails could be cryopreserved and still retained full CD83 expression after thawing. However, some loss of viability was noted, particularly with DC2 and DC5 cells matured in media containing poly (I:C).

Improved understanding of DC biology has led to continually evolving ideas regarding optimal DC-based vaccines. The mere replacement of iDCs with phenotypically mDCs, expressing high levels of MHC and costimulatory molecules alongside the CCR7 homing receptor, must now be extended by considerations regarding the various roles that DCs play based on their cytokine secretion patterns [42]. This consideration is reflected here by the different ratios of IL-12p70/IL-10 in DC1 (Jonuleit) cocktail versus DC2 (Kalinski) cocktail. Such variations would have the consequence of yielding DC vaccines with different polarizing capacities. On one hand, immune responses primed by some DCs could lead to induction of regulatory cells that could be useful for transplant tolerance or autoimmunity, whereas others could induce Th1-polarized immune responses desirable for optimal anti-tumor immunity or Th2-directed responses that are essential for antibody responses critical for some infectious diseases. DC-based vaccines that are generated *in vitro *must also have the capacity to secrete cytokines upon contact with T cells *in vivo*. A first assessment of cytokine capacity was made using supernatant medium from primary DCs. However, the signal-3 assay was important to evaluate induction of cytokine secretion in a setting mimicking a T cell encounter, with subsequent DC signalling via CD40L, in order to demonstrate that the DCs were not exhausted with respect to cytokine production [35]. IL-12p70 secretion by DCs is stimulus dependent and underlies a short kinetic [43]. Results of the signal-3 assay revealed that DCs matured in cocktails DC2 and DC5 showed the highest levels of IL-12p70 secretion and DC2–DC5 cells were all superior to DC1 cells with respect to IL-12p70/IL-10 ratios.

When all characteristics were taken together, DCs matured with our cocktails, particularly cocktails 3 and 4, combined the best characteristics of DC1 (Jonuleit) cocktail with respect to phenotype, maturation stability, function and recovery after cryopreservation, combined with the additional capacity to produce IL-12p70. These findings correlate with recent observations that inflammatory cytokines alone are not able to induce full DC maturation [18], but rather that DCs also require signals through TLR or other innate, non-TLR pattern recognition receptors, representing nature's way of engaging pathogens through pattern recognition. The presence of the synthetic TLR8 ligand R848 in our media correlated with DC2 (Kalinski) cocktail with respect to an IL-12-inducing capacity due to a TLR-induced autocrine type 1 interferon loop [44]. The cytotoxic property of IFNα, present in DC2 cocktail, may have contributed to poor DC viability. Viability may have been further impacted by the presence of poly (I:C), which signals through distinct pathways, including endosomal TLR3 but mainly via RNA-sensing helicase domain-containing proteins within the cytosol [45,46]. Endosomal TLR7/8 signalling by R848[41,47] showed no negative impact on cell viability, at least not at the concentration of 1 μg/ml used here. In contrast to Napolitani et al. [39], we presume that synergistic stimulation through poly (I:C) and R848, may lead to over-stimulation of DCs with some induction of apoptosis, explaining the lower viability of DCs matured in cocktail DC5.

Furthermore, we found that the addition of poly (I:C) to the basic components of cocktail DC4, creating the DC5 cocktail, prevented DC5 cells from being able to express protein after loading with exogenous RNA, presumably through induction of mechanisms to protect cells from foreign RNA [47]. This restriction of RNA expression was similar to the behaviour of DCs matured in DC2 (Kalinski) cocktail, which also contained poly (I:C). Thus, DCs matured in medium containing poly (I:C) appear unsuitable for use in RNA-based vaccines, although both are clearly suitable for use with peptides, as shown here for cocktail DC5 and published previously for DC2 (Kalinski) cocktail [20]. In contrast, DCs matured in cocktails DC3 and DC4 would be well suited for generating IL-12p70-producing mDCs for cancer vaccine development using either peptides or RNA as sources of tumor-associated antigens.

## Conclusion

Here we describe new maturation cocktails that can be used to generate human monocyte-derived dendritic cells with IL-12p70-secreting potential after CD40L stimulation. These mDCs show good harvesting characteristics and their generation can be easily adapted for compliance with good manufacturing practice. Our preferred cocktail is the DC4 cocktail, which combines the best characteristics of the current "gold standard" DC1 (Jonuleit) cocktail and the recently published alternative DC2 (Kalinski) cocktail. DCs prepared with cocktail DC4 are suitable for use with RNA, in addition to peptides, as a source of tumor-associated antigens.

## Abbreviations

CCR: chemokine receptor (C-C type)

CD: cluster of differentiation

CEF: CMV, EBV, influenza virus

DCs: dendritic cells

DTH: delayed type of hypersensitivity

EGFP: enhanced green fluorescent protein

FACS: fluorescence activated cell sorting

GMP: good manufacturing practice

GM-CSF: granulocyte macrophage-colony stimulating factor

iDCs: immature dendritic cells

IFN: interferonIL: interleukin

mDCs: mature dendritic cells

MHC: major histocompatibility complex

MLR: mixed lymphocyte reaction

PBS: phosphate buffered saline

PG: prostaglandin

PHA: phytohaemaglutinin

poly (I:C): polyriboinosinic:polyribocytidylic acid

RPMI: Rosewell Park Memorial Institute – cell culture medium

TGF: transforming growth factor

Th: T helper cell

TNF: tumor necrosis factor

TLR: toll-like receptor

## Competing interests

The authors declare a competing interest through a pending patent, submitted by the GSF National Research Center for Environment and Health, Munich, Germany, for these newly described maturation cocktails to generate clinically applicable DCs.

## Authors' contributions

**AZ **developed the new maturation cocktails, established the ELUTRA system and performed experiments to generate DCs, analyzed DC phenotypes, performed signal-3 assays, ELISAs, proliferation assays, freezing protocols, prepared data and drafted the manuscript.

**MJ **performed the experiments on RNA transfer into DCs and their analysis by flow cytometry and created the final figures for the manuscript.

**BF **helped with the analyses of IL-12 and with the preparation of the manuscript.

**HP **performed the ELISPOT experiments to detect IFNγ induced by different DC subpopulations, including data assessment.

**EK **established rat hybridoma BLR2 (clone 8E8) against CCR7 and the isotype control hybridoma (EBNA-A2 clone R3), provided supernatants and advised on the use of secondary antibody for FACS analysis.

**IB **advised on GMP compliance for applications of ELUTRA and Coulter Counter ACT Dif.

**DJS **provided scientific advice, discussions of data and contributed to the writing and revisions of the manuscript.
